# ﻿Establishment of two new *Anaplecta* species (Blattodea, Blattoidea, Anaplectidae) based on morphological and *COI* data with an additional description of *Anaplectafurcata* Deng & Che, 2020

**DOI:** 10.3897/zookeys.1130.87810

**Published:** 2022-11-21

**Authors:** Xinyi Deng, Jieyu Shan, Chengcheng Xiao, Jing Zhu, Zongqing Wang, Yanli Che

**Affiliations:** 1 College of Plant Protection, Southwest University, Beibei, Chongqing 400715, China Southwest University Beibei China

**Keywords:** Cockroaches, *COI*, DNA barcodes, female genitalia, morphology, new species, taxonomy

## Abstract

Based on morphological characteristics, including male and female genitalia, combined with DNA barcodes, two new species, *Anaplectacircinalis* Deng & Che, **sp. nov.** and *Anaplectabihamata* Deng & Che, **sp. nov.**, are described in detail. Additional information on the female genitalia of *Anaplectafurcata* Deng & Che, 2020 is also provided. Photographs of external morphology and caudal anatomy of these species, as well as a key to the Chinese *Anaplecta* species, are provided.

## ﻿Introduction

At present, 112 species of *Anaplecta* have been recorded, widely distributed in Asia, North America, South America, Africa and Oceania (21 species in China) (Beccaloni 2014; Deng et al. 2020; Zhu et al. 2022). As taxonomic research progresses, both morphological characters including male and female genitalia and DNA barcodes have been applied to the identification of *Anaplecta* species (Deng et al. 2020; Zhu et al. 2022). This has enriched the knowledge of the *Anaplecta* fauna, which shows a rich diversity.

The effective discernment of species using male genitalia was verified in Deng et al. (2020) although subtle intraspecific variation occurred in male genitalia of some *Anaplecta* species. Three cryptic *Anaplecta* species were revealed in Zhu et al. (2022), where they combined male and female genitalia with DNA barcodes. This confirmed the importance of female genitalia in species delimitation of this genus.

Recently, after collecting cockroach specimens in Fujian, Yunnan, Guangdong and Hunan provinces in China, two new *Anaplecta* species were discovered based on morphological characters. This result was then further verified here by using DNA barcodes. In addition, an exhaustive description of the female genitalia of *Anaplectafurcata* was also provided herein.

## ﻿Materials and methods

### ﻿Morphological study

Sixty-eight specimens from Fujian, Yunnan, Guangdong and Hunan provinces were examined in this study. The genitalia terminology used in this paper mainly follows McKittrick (1964), Roth (1990) and Deng et al. (2020), while veins terminology follows Li et al. (2018).

The measurements are based on the specimens examined. The genitalia were processed with 10% NaOH at 65 °C for 30–35 min for digestion of soft tissues. The genitalia segments were dissected and stored in glycerol, then observed with a Motic K400 stereomicroscope. These segments were then preserved with the remainder of the specimen in ethyl alcohol. Photographs of the genitalia and body were taken with a Leica M205A stereomicroscope and edited with Adobe Photoshop CS6. All type materials are deposited at the Institute of Entomology, College of Plant Protection, Southwest University, Chongqing, China (SWU).

Abbreviations in this paper are as follows:

**L1, L2, L3** sclerites of the left phallomere;

L2dL2 dorsal;

**L2v**L2 ventral;

**L2vm** median sclerite;

**M** media veins;

**R1, R2, R3** sclerites of the right phallomere;

**CuA** cubitus anterior;

**CuP** cubitus posterior.

### ﻿PCR amplification and sequencing

Four specimens were used for cytochrome oxidase subunit I (*COI*) sequencing in this study. Total DNA was extracted from the muscle of legs according to the HiPure Tissue DNA Mini Kit (Magen Biotech, Guangzhou). Primers for polymerase chain reaction (PCR) were *COI*-F (5’-CAACYAATCATAAAGANATTGGAAC-3’) and *COI*-R (5’-TAAACTTCTGGRTGACCAAARAATCA-3’) (a simple adjustment based on Folmer 1994; Yang et al. 2019). The amplification conditions were as follows: initial denaturation 2 min at 98 °C, followed by 35 cycles of 10 s at 98 °C, 10 s annealing at 49–50 °C, 15 s extension at 72 °C, and a final extension of 2 min at 72 °C; the samples were then held at 8 °C. The PCR products were sequenced by Tsingke Biotechnology Co., Ltd. (Beijing, China).

### ﻿Molecular analyses

A total of 62 *COI* sequences were analyzed in this study: four newly-sequenced sequences of our newly described *Anaplecta* species and 55 published sequences of 20 Chinese *Anaplecta* species, and three sequences of *Periplaneta* Burmeister, 1838 (as outgroup) downloaded from GenBank (Table 1). The alignment was then manually corrected by translation into amino acids in MEGA 7 (Kumar et al. 2016). The genetic divergence value was quantified based on the Kimura 2-parameter (K2P) distance model (Kimura 1980). Maximum likelihood (ML) analysis was implemented in IQ-TREE (Nguyen et al. 2015) with 1000 replicates for bootstrap values, after choosing optimal partitioning scheme and substitution models (*COI*_pos 1, GTR+I+G; *COI*_pos 2 and *COI*_pos 3, HKY+I+G) in PartitionFinder v.2.1.1 (Lanfear et al. 2017) with the corrected Akaike Information Criterion (AICc).

**Table 1.** Samples of *Anaplecta* species used in the maximum likelihood analyses.

Species	Location (voucher number, gender)	Literature source	GenBank Accession Number
*A.circinalis* sp. nov.	Pu’er, Yunnan (Anapcircm, ♂)		OP306078
*A.circinalis* sp. nov.	Pu’er, Yunnan (Anapcircf, ♀)		OP306077
*A.bihamata* sp. nov.	Shaoyang, Hunan (Anapbiham, ♂)		OP306076
*A.bihamata* sp. nov.	Shaoyang, Hunan (Anapbihaf, ♀)		OP306075
*A.bicruris*		Zhu et al. (2022)	OL790029, OL790030, OL790036
*A.spinosa*		Zhu et al. (2022)	OL790028, OL790038
*A.ungulata*		Zhu et al. (2022)	OL790031, OL790053, OL790048
*A.anomala*		Zhu et al. (2022)	OL790032, OL790050
*A.serrata*		Zhu et al. (2022)	OL790033, OL790047, OL790046
*A.bombycina*		Zhu et al. (2022)	OL790037, OL790049, OL790034, OL790052
*A.longihamata*		Zhu et al. (2022)	OL790035, OL790051
*A.paraomei*		Zhu et al. (2022)	OL790039, OL790045, OL790041, OL790042
*A.condensa*		Zhu et al. (2022)	OL790040, OL790043, OL790044
*A.truncatula*		Zhu et al. (2022)	OL790054, OL790055
*A.omei*		Zhu et al. (2022)	OL790056, OL790057, OL790058
*A.omei*		Deng et al. (2020)	MT800287
*A.corneola*		Zhu et al. (2022)	OL790063
*A.corneola*		Deng et al. (2020)	MT800293, MT800296
*A.cruciata*		Zhu et al. (2022)	OL790061
*A.cruciata*		Deng et al. (2020)	MT800303, MT800304
*A.basalis*		Zhu et al. (2022)	OL790060
*A.basalis*		Deng et al. (2020)	MT800305, MT800309
*A.nigra*		Deng et al. (2020)	MT800306
*A.staminiformis*		Zhu et al. (2022)	OL790062
*A.staminiformis*		Deng et al. (2020)	MT800297, MT800299
*A.arcuata*		Zhu et al. (2022)	OL790065
*A.arcuata*		Deng et al. (2020)	MT800307, MT800308
*A.strigata*		Zhu et al. (2022)	OL790064
*A.strigata*		Deng et al. (2020)	MT800291, MT800292
*A.furcata*		Deng et al. (2020)	MT800301,MT800302
*A.bicolor*		Zhu et al. (2022)	OL790059
*A.bicolor*		Deng et al. (2020)	MT800310
*Periplanetaamericana*		Jones et al. (2013)	KC617846
*Periplanetafuliginosa*		Beeren et al. (2015)	KM577133
*Periplanetaaustralasiae*		Yue et al. (2014)	KF640069

## ﻿Results

### ﻿Morphological delimitation based on external morphology and genitalia

After observing the external morphological and genital characteristics of 42 *Anaplecta* samples from Fujian, Yunnan, Guangdong and Hunan provinces, two new morphospecies and one known species, *Anaplectafurcata*, were identified. One morphospecies can be distinguished by its curled L2vm from other Chinese *Anaplecta* species; while the other is characterized by its hook-shaped L2vm and R1.

### ﻿Molecular analysis based on *COI*

**Figure 1.**
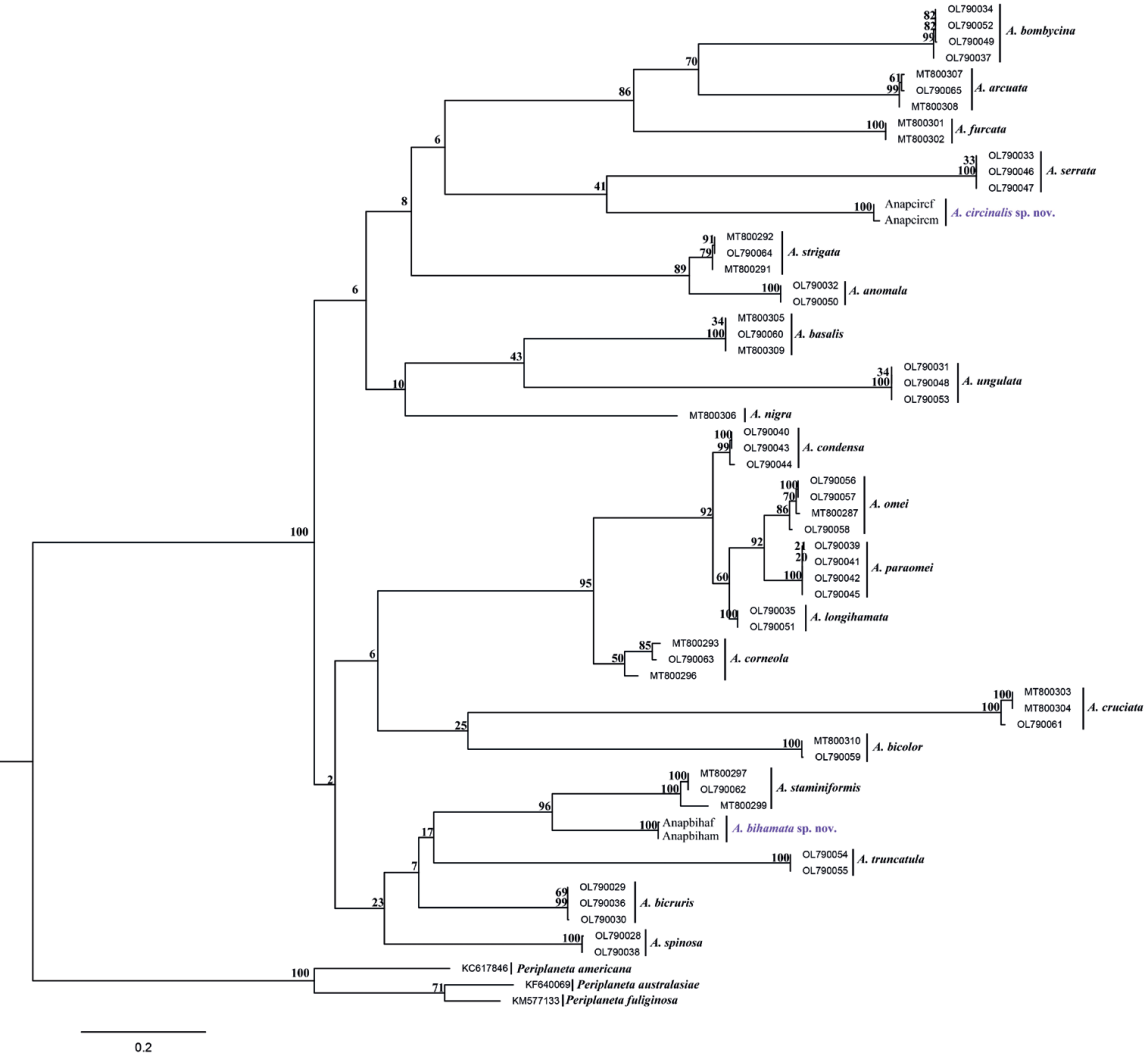
Maximum likelihood (ML) tree derived from *COI* sequences implemented in IQ-TREE with 1000 replicates for bootstrap values.

In this study, the sequenced length of *COI* excluding the primer was approximately 658bp. Four new *COI* sequences have been deposited in GenBank with accession numbers OP306075 to OP306078 (Table 1). Interspecific *COI* genetic divergence ranged from 5.54% (*A.longihamata* and *A.condensa*) to 27.53% (*A.truncatula* and *A.ungulata*) (Table 2). Interspecific *COI* genetic divergence ranges among the two new morphospecies (*A.bihamata* sp. nov. and *A.circinalis* sp. nov.) and other *Anaplecta* species are 13.97–27.53%, and 18.85–24.85% respectively (Table 2). ML analysis revealed that conspecific samples including two new morphospecies (*A.bihamata* sp. nov. and *A.circinalis* sp. nov.) gathered together well to constitute monophyletic groups (Fig. 1), which solidly supported our morphological results.

**Table 2.** Interspecific genetic distance calculated by the K2P model using *COI* sequences in MEGA.

	species	1	2	3	4	5	6	7	8	9	10	11	12	13	14	15	16	17	18	19	20	21
**1**	***A.bihamata* sp. nov.**																					
**2**	*A.staminiformis*	0.1397																				
**3**	*A.bicruris*	0.1686	0.1827																			
**4**	*A.spinosa*	0.1835	0.1764	0.1653																		
**5**	*A.longihamata*	0.2177	0.2460	0.2134	0.2222																	
**6**	*A.condensa*	0.2121	0.2370	0.2160	0.2131	**0.0554**																
**7**	*A.paraomei*	0.2003	0.2220	0.2037	0.2169	0.0832	0.0881															
**8**	*A.omei*	0.1966	0.2202	0.2192	0.2065	0.0779	0.0937	0.0699														
**9**	*A.corneola*	0.2078	0.2115	0.1972	0.1975	0.1425	0.1447	0.1437	0.1513													
**10**	*A.nigra*	0.1899	0.1904	0.1943	0.2150	0.2318	0.2365	0.2146	0.2277	0.2108												
**11**	*A.basalis*	0.1734	0.1875	0.1918	0.1909	0.2417	0.2400	0.2176	0.2202	0.2133	0.2132											
**12**	*A.bicolor*	0.2265	0.2215	0.1939	0.2025	0.2211	0.2209	0.2104	0.2207	0.2070	0.2399	0.2246										
**13**	*A.anomala*	0.2123	0.2119	0.2159	0.2065	0.2106	0.2154	0.1981	0.1974	0.2157	0.2220	0.2342	0.1972									
**14**	*A.strigata*	0.2123	0.2236	0.2077	0.2012	0.2032	0.2100	0.2095	0.2035	0.2004	0.2094	0.2104	0.2071	0.0868								
**15**	*A.serrata*	0.2027	0.2287	0.2090	0.2198	0.2366	0.2526	0.2213	0.2144	0.2353	0.2141	0.2124	0.2438	0.1986	0.1852							
**16**	***A.circinalis* sp. nov.**	0.2014	0.2175	0.2179	0.1885	0.2427	0.2485	0.2236	0.2196	0.2416	0.2368	0.2063	0.2282	0.2145	0.2019	0.2051						
**17**	*A.bombycina*	0.2487	0.2423	0.2201	0.2050	0.2269	0.2310	0.2247	0.2198	0.2141	0.2176	0.2530	0.2471	0.2263	0.2216	0.2373	0.2263					
**18**	*A.arcuata*	0.1970	0.2339	0.2367	0.2310	0.2258	0.2355	0.2197	0.2291	0.2442	0.2128	0.2364	0.2239	0.2089	0.2100	0.2495	0.2027	0.1925				
**19**	*A.furcata*	0.2201	0.2357	0.2133	0.2208	0.2199	0.2419	0.2376	0.2178	0.2284	0.2126	0.2275	0.2128	0.2124	0.2084	0.2059	0.2192	0.1887	0.1867			
**20**	*A.truncatula*	0.2025	0.2161	0.2008	0.1961	0.2387	0.2274	0.2187	0.2219	0.2400	0.2395	0.2031	0.2393	0.2405	0.2398	0.2348	0.2099	0.2494	0.2456	0.2334		
**21**	*A.cruciata*	0.2087	0.2167	0.2201	0.2258	0.2352	0.2233	0.2359	0.2107	0.2475	0.2291	0.2292	0.2354	0.2228	0.2370	0.2383	0.2230	0.2615	0.2342	0.2362	0.2546	
**22**	*A.ungulata*	0.2337	0.2644	0.2720	0.2411	0.2424	0.2503	0.2509	0.2536	0.2349	0.2284	0.2250	0.2569	0.2276	0.2068	0.2628	0.2435	0.2504	0.2374	0.2454	**0.2753**	0.2571

### ﻿Taxonomy

Based on the morphology and ML analysis, we confirmed two new species: *Anaplectacircinalis* Deng & Che, sp. nov. and *Anaplectabihamata* Deng & Che, sp. nov.

### ﻿Key to species of *Anaplecta* in China

[*A.simplex* Shiraki, 1931 is not included because we were unable to collect a sample and only wings were described by the author.]

**Table:** 

1	Disk of pronotum bicolored	**2**
–	Disk of pronotum unicolored	**6**
2	Disk dark and faded gradually or sharply towards the posterior end	**3**
–	Disk dark with some markings	**4**
3	Tegmina yellowish brown, 1/3 of the base black (except the lateral margins)	***A.basalis* Bey-Bienko, 1969**
–	Tegmina completely yellowish brown (except the lateral margins)	***A.bicolor* Deng & Che, 2020**
4	Disk with a pair of blurred longitudinal darker areas	***A.bicruris* Zhu & Che, 2022**
–	Disk with a lighter blurred centre	**5**
5	Tegmina unicolored	***A.strigata* Deng & Che, 2020**
–	Tegmina bicolored, 1/3 of the base darker than remaining parts (except lateral margins and anal field)	***A.anomala* Zhu & Che, 2022**
6	Tegmina with obvious markings	**7**
–	Tegmina without obvious markings	**9**
7	Tegmina yellowish brown, with a nearly oval brown spot at CuP	***A.ungulata* Zhu & Che, 2022**
–	Tegmina yellowish brown, with a subrectangular black spot at base	**8**
8	R1 needle-shaped	***A.truncatula* Zhu & Che, 2022**
–	R1 arc-shaped	***A.nigra* Deng & Che, 2020**
9	Male paraprocts with dense spines on curly posterior margin	**10**
–	Male paraprocts not as above	**13**
10	Intercalary sclerite small, nearly filamentous	***A.condensa* Zhu & Che, 2022**
–	Intercalary sclerite large, strip-shaped or sheet-like	**11**
11	Right first valvifer arm long, lateral edges folded up	***A.longihamata* Zhu & Che, 2022**
–	Right first valvifer arm short, lateral edges not folded up	**12**
12	The posterior margin of anterior arch hip-shaped	***A.paraomei* Zhu & Che, 2022**
–	The posterior margin of anterior arch smooth	***A.omei* Bey-Bienko, 1958**
13	L1 with a long and curved filamentary structure	**14**
–	L1 with a short and robust uncinate structure	***A.cruciata* Deng & Che, 2020**
14	R1 degraded or merged with L2vm	**15**
–	R1 well developed, not merged with L2vm	**18**
15	Male paraprocts specialized, stripe-shaped, with spines on posterior margin	***A.spinosa* Zhu & Che, 2022**
–	Male paraprocts unspecialized	**16**
16	The apex of L2v bifurcated, sheet-like	**17**
–	The apex of L2v not bifurcated, shaped like ‘3’	***A.bombycina* Zhu & Che, 2022**
17	One sclerite of R2 serrated	***A.serrata* Zhu & Che, 2022**
–	All sclerites of R2 without serration	**18**
18	L2vm slender	***A.arcuata* Deng & Che, 2020**
–	L2vm broad	***A.circinalis* Deng & Che, sp. nov.**
19	R1 curved	**20**
–	R1 straight, cylindrical	***A.staminiformis* Deng & Che, 2020**
20	R1 highly sclerotized, horn-shaped	**21**
–	R1 sightly sclerotized, arc-shaped	***A.furcata* Deng & Che, 2020**
21	Right phallomere without special horny structure	***A.bihamata* Deng & Che, sp. nov.**
–	Right phallomere with special horny structure	***A.corneola* Deng & Che, 2020**

#### ﻿ Anaplecta circinalis

Taxon classificationAnimaliaBlattodeaAnaplectidae

Deng & Che sp. nov.

FA07A459-CCA6-5693-8EB6-B40DEC61A517

https://zoobank.org/90ED9DF4-AEE9-4927-8EE8-835ACD03312A

Figs 2A–I , 4A–C

##### Type material.

***Holotype***: China • male; Yunnan Prov., Pu’er County, Xiniuping Scenic Area of Pu’er National Park; 26°36.14'N, 101°5.53'E; 1602 m; 29 Jun. 2021; Jia-Wei Zhang & Jin-Lin Liu leg; SWU-B-AN-0175. ***Paratypes***: China • 9 males & 14 females; same data as holotype; SWU-B-AN-0176 to 0198.

**Diagnosis.** This species can be easily separated from other Chinese *Anaplecta* species by its curled left phallomere (L2vm).

##### Etymology.

The specific epithet ‘*circinalis*’ is derived from the Latin word *circinalis*, referring to the curled L2vm.

##### Description.

***Measurements* (mm)**. **Male**: Pronotum length × width: 1.0–1.3 × 1.8–1.9, tegmina length: 5.0–5.2, overall length: 5.8–6.1. **Female**: Pronotum length × width: 1.2–1.4×1.7–2.0, tegmina length: 4.7–5.1, overall length: 5.6–6.2.

**Figure 2.**
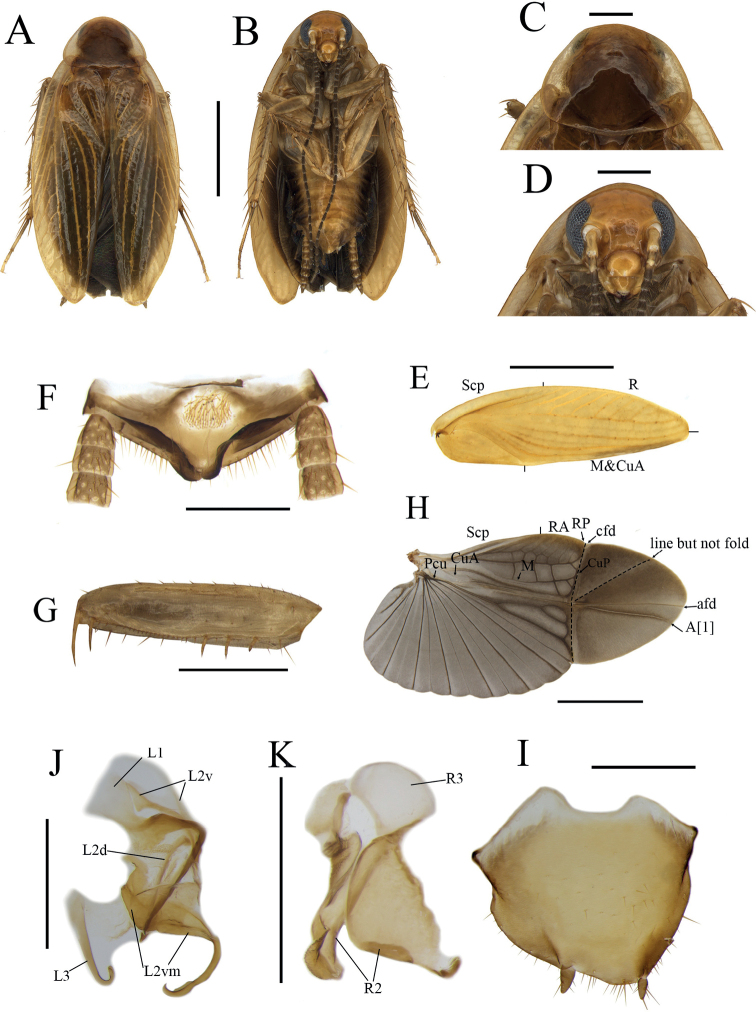
*Anaplectacircinalis* Deng & Che, sp. nov. holotype, male SWU-B-AN-0175 **A** habitus, dorsal view **B** habitus, ventral view **C** pronotum, dorsal view **D** head, ventral view **E** tegmen **F** supra-anal plate, ventral view **G** front femur **H** wing **I** subgenital plate, dorsal view **J** left phallomere **K** right phallomere, dorsal view. Abbreviations: **afd** anal fold, **A[1**] anterior anal vein, **cfd** cubitus fold, **CuA** cubitus anterior, **CuP** cubitus posterior, **L1, L2, L3** sclerites of the left phallomere, **L2d**L2 dorsal, **L2v**L2 ventral, **L2vm** median sclerite, **M** median, **Pcu** postcubitus, **R** radius, **RA** radius anterior, **RP** radius posterior, **R2, R3** sclerites of the right phallomere, **ScP** subcostal posterior. Scale bars: 2 mm (**A–E, G, H**); 0.5 mm (**F, I–K**).

***Coloration*.** Body dark yellowish brown, eyes black, antennae dark brown. Head yellowish brown (Fig. 2A, B, D). Pronotum dark yellowish brown, lateral borders nearly hyaline (Fig. 2C). Tegmina yellowish brown, lateral borders nearly hyaline (Fig. 2E). Wings with costal field and appendicular field light brown, other parts light brown, veins dark brown (Fig. 2H). Center of abdominal sterna yellowish brown, gradually darkening to edges. Cerci and legs yellowish brown.

***Head and thorax*.** Interocular space slightly greater than distance between antennal sockets. Third and fifth maxillary palpi equal in length, longer than fourth; fifth maxillary palpus subelliptical and thicker than other segments (Fig. 2D). Pronotum subelliptical, anterior margin arched, hind margin nearly straight (Fig. 2C).

Tegmina with indistinct veins; the radius posterior veins of hind wings slightly indistinct, CuP and CuA merging into one venation (Fig. 2E, H). Front femur type B_2_ (Fig. 2G), pulvilli absent, tarsal claws symmetrical, arolia present.

***Male abdomen and genitalia*.** Supra-anal plate nearly symmetrical, sheet-like (Fig. 2F). Subgenital plate asymmetrical, interstylar margin arched, with a pair of anterior extensions (Fig. 2I). The left styli more robust than the right. Styli with length about 1/3 of interstylar space (Fig. 2I). Phallomere complex, L1 small, with slender and curved filamentary structure. L2v elongated, slightly bifurcated. L2vm broad, slightly thickened and curved. L2d slender and bifurcated. L3 slender, uncinate part blunt (Fig. 2J). R1 absent. R2 irregular, sheet-like, slightly sclerotized. R3 simple, sheet-like (Fig. 2K).

***Female abdomen and genitalia*.** Supra-anal plate nearly symmetrical. Paraprocts (pp.) broad, not extending to the posterior margin of supra-anal plate. Intercalary sclerite (intc.s) nearly stripe-shaped, slightly curved. First valvifer arm short. First valve (v.I) robust. Second valve (v.II) small, basally fused. Third valve (v.III) broad. The anterior margin of anterior arch (aa.) symmetrical, slightly sclerotized, extending forward in a flaky shape with a deep concavity in the middle. Basivalvula (bsv.) nearly flattened, elliptical (Fig. 4A, B). Laterosternal shelf (ltst.sh) slightly sclerotized, lateral margin straight (Fig. 4C).

##### Distribution.

China (Yunnan).

#### ﻿ Anaplecta bihamata

Taxon classificationAnimaliaBlattodeaAnaplectidae

Deng & Che sp. nov.

FF97676B-3712-5C0F-AE1E-312A79335A0C

https://zoobank.org/01866CE9-D683-4CB2-94C1-5DB812542B74

Figs 3A–I , 4D–F

##### Type material.

***Holotype***: China • male; Hunan Prov., Shaoyang City, Chengbu County, Ten Miles Flat Monitoring Station; 26°14.12'N, 110°25.52'E; 821 m; 22 May 2021; Jing Zhu leg; SWU-B-AN-0199. ***Paratypes***: China • 7 males; SWU-B-AN-0200 to 206; same collection data as holotype • 1 male; SWU-B-AN-0207; Guangdong Prov., Shaoguan City • 2 males and 1 female; SWU-B-AN-0208 to 0210; Ruyuan County, Nanling Nature Reserve Xiaozhu Parking Lot; 24°54.10'N, 113°2.53'E; 695 m, 18 May 2021; Wei Han & Li-Min Qiao leg. • 1 male; SWU-B-AN-0211; Hunan Prov., Yongzhou City, Ningyuan County, Mt. Jiuyi, Yellow River Village; 25°9.8'N, 111°34.17'E; 629 m, 6 Jun. 2021; Jing Zhu leg. • 3 males; SWU-B-AN-0212 to 214; Fujian Prov., Wuyishan City, Sisin Integrated Observation Site; 27°35.30'N, 117°46.4'E; 450 m, 23 Jun. 2021; Wei Han & Jing Zhu leg.

##### Diagnosis.

This species can be easily separated from other Chinese *Anaplecta* species by its hook-shaped L2vm and R1.

##### Etymology.

The specific epithet is derived from the Latin word *hamatus*, referring to both L2vm and R1 being hook-like.

##### Description.

***Measurements* (mm)**. **Male**: Pronotum length × width: 1.2–1.4 × 1.9–2.0, tegmina length: 5.8–6.4, overall length: 6.8–7.3. **Female**: Pronotum length × width: 1.1–1.4 × 1.9–2.2, tegmina length: 5.4–5.6, overall length: 6.2–6.7.

**Figure 3.**
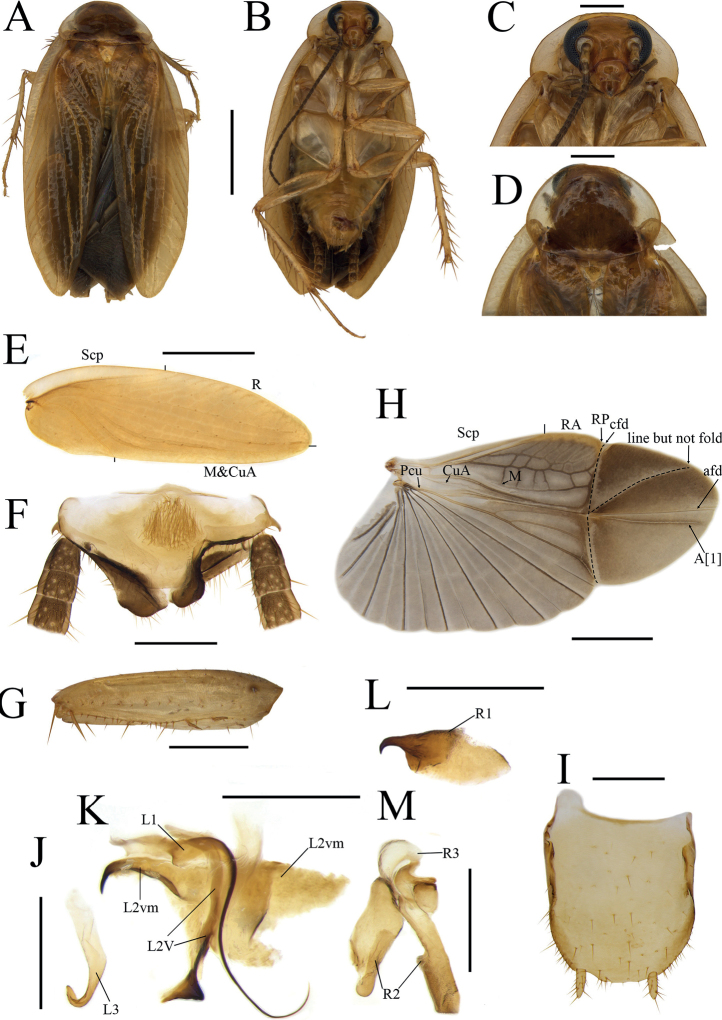
*Anaplectabihamata* Deng & Che, sp. nov. holotype, male SWU-B-AN-0177 **A** habitus, dorsal view **B** habitus, ventral view **C** pronotum, dorsal view **D** head, ventral view **E** tegmen **F** supra-anal plate, ventral view **G** front femur **H** wing **I** subgenital plate, dorsal view **J, K** left phallomere **L, M** right phallomere, dorsal view. Abbreviations: **afd** anal fold, **A[1**] anterior anal vein, **cfd** cubitus fold, **CuA** cubitus anterior, **CuP** cubitus posterior, **L1, L2, L3** sclerites of the left phallomere, **L2v**L2 ventral, **L2vm** median sclerite, **M** median, **Pcu** postcubitus, **R** radius, **RA** radius anterior, **RP** radius posterior, **R1, R2, R3** sclerites of the right phallomere, **ScP** subcostal posterior. Scale bars: 2 mm (**A–E, G, H**); 0.5 mm (**F, I–M**).

***Coloration*.** Body yellowish brown, eyes black, antennae dark brown. Head yellowish brown (Fig. 3A, B, C). Pronotum and tegmina yellowish brown, lateral borders nearly hyaline (Fig. 3D, E). Wings with costal field and appendicular field infuscated, other parts light brown, with veins dark brown (Fig. 3E, H). Abdominal sterna, cerci and legs yellowish brown (Fig. 3B).

***Head and thorax*.** Interocular space slightly greater than distance between antennal sockets. Fourth and fifth maxillary palpi equal in length, shorter than third maxillary palpus; fifth maxillary palpus triangular and thicker than others (Fig. 3C). Pronotum subelliptical (Fig. 3D). Tegmina with indistinct veins; wings with radial veins slightly indistinct, CuP and CuA merging into one venation (Fig. 3E, H). Front femur type B_2_ (Fig. 3G), pulvilli absent, tarsal claws symmetrical, arolia present.

***Male abdomen and genitalia*.** Supra-anal plate symmetrical, sheet-like (Fig. 3F). Subgenital plate subelliptical, with an anterior extension in the left and the posterior margin slightly arched (Fig. 3I). Styli small, cylindrical, styli with length about ⅓ of interstylar space (Fig. 3I). Phallomere complex, L1 small, with slender and curved filamentary structure. L2v elongated, bifurcated at apex and highly sclerotized at terminal. L2vm broad with curved hook at the left (Fig. 3K). L3 robust and medium, uncinate part with apex blunt (Fig. 3J). R1 hooked, the proximal part sharply tapered and highly sclerotized (Fig. 3L). R2 irregular, slightly sclerotized. R3 short, simple sheet-like (Fig. 3M).

**Figure 4.**
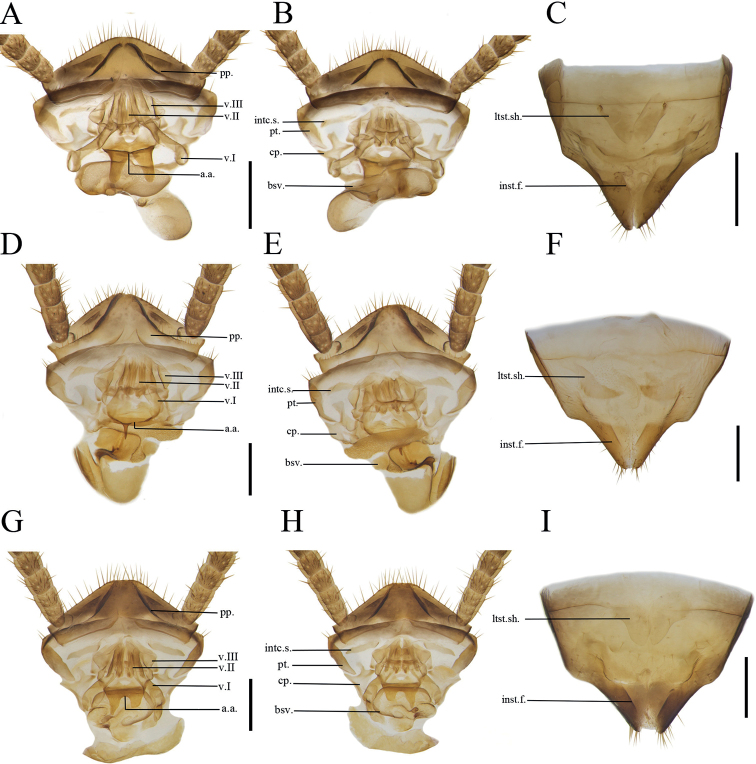
**A–C***Anaplectacircinalis* Deng & Che, sp. nov. paratype, female SWU-B-AN-0176 **D–F***Anaplectabihamata* Deng & Che, sp. nov. paratype, female SWU-B-AN-0178 **G–I***Anaplectafurcata* female SWU-B-B-A060471 **A, D, G** supra-anal plate, ventral view **B, E, H** supra-anal plate, dorsal view **C, F, I** subgenital plate, dorsal view. Abbreviations: **a.a.** anterior arch, **bsv.** basivalvula, **cp.** crosspiece, **pt**. paratergites, **intc.s.** intercalary sclerite, **inst.f.** Intersternal fold, **ltst.sh.** laterosternal shelf, **pp.** paraprocts, **sp.** spermatheca, **v.I** first valve, **v.II** second valve, **v.III** third valve. Scale bars: 2 mm.

***Female abdomen and genitalia*.** Supra-anal plate nearly symmetrical. Paraprocts (pp.) broad, extending to the posterior margin of supra-anal plate. Intercalary sclerite (intc.s) short, nearly stripe-shaped, slightly curved. First valvifer arm short. First valve (v.I) robust. Second valve (v.II) small, basally fused. Third valve (v.III) broad. The anterior margin of anterior arch (aa.) slightly sclerotized, with a near spine-shaped protrusion and dense tiny punctuations. Basivalvula (bsv.) nearly flat (Fig. 4D, E). Laterosternal shelf (ltst.sh) slightly sclerotized lateral margin straight (Fig. 4F).

##### Distribution.

China (Hunan, Fujian, Guangdong).

#### ﻿ Anaplecta furcata

Taxon classificationAnimaliaBlattodeaAnaplectidae

Deng & Che, 2020

A2D25AA9-94F7-5569-88F2-6B3F2005F76C

Fig. 4G–I

Anaplecta furcata Deng & Che in Deng et al. 2020: 93–95.

##### Description.

***Measurements* (mm)**. **Female**: pronotum length × width: 1.1–1.4 × 1.9–2.2, tegmina length: 5.4–5.6, overall length: 6.2–6.7.

***Female abdomen and genitalia*.** Supra-anal plate nearly symmetrical. Paraprocts broad, extending to the posterior margin of supra-anal plate. Intercalary sclerite (intc.s) nearly stripe-shaped. First valve (v.I) robust. Second valve (v.II) small, basally fused. Third valve (v.III) broad. The anterior margin of anterior arch (aa.) slightly sclerotized, extending forward in a flaky shape with a deep concavity in the middle. Basivalvula (bsv.) broad, some areas with dense punctuations. Laterosternal shelf (ltst.sh) slightly sclerotized, lateral margin slightly curved.

##### Material examined.

China • 2 females; SWU-B-AN-0215 to 216; Guangxi Prov., Jinxiu County, Mt. Dayao; 24°8.43'N, 110°11.70'E; 944 m; 7 Jul. 2015; Lu Qiu & Qi-Kun Bai leg.

##### Distribution.

China (Guangxi).

## Supplementary Material

XML Treatment for Anaplecta circinalis

XML Treatment for Anaplecta bihamata

XML Treatment for Anaplecta furcata

## References

[B1] Beccaloni GW (2014) Cockroach Species File Online. Version 5.0/5.0. http://cockroach.speciesfile.org/ [accessed 22 May 2022]

[B2] Beeren CV Stoeckle MY Xia J Burke G Kronauer DJ (2015) Interbreeding among deeply divergent mitochondrial lineages in the American cockroach (*Periplanetaamericana*). Scientific Reports 5(1): 8297. 10.1038/srep08297 25656854 PMC4650827

[B3] Bey-Bienko GY (1958) Results of the Chinese-Soviet Zoological-Botanical Expeditions of 1955–56 to southwestern China. Blattoidea of Szechuan and Yunnan II. Entomological Review 582–597. 10.11646/zootaxa.5099.2.7

[B4] Bey-Bienko GY (1969) New genera and species of cockroaches (Blattoptera) from tropical and subtropical Asia. Entomologicheskoe Obozrenie 48: 831–862. 10.11646/zootaxa.4532.4.4

[B5] Deng WB Liu YC Wang ZQ Che YL (2020) Eight new species of the genus *Anaplecta* Burmeister, 1838 (Blattodea: Blattoidea: Anaplectidae) from China based on molecular and morphological data. European Journal of Taxonomy 720: 77–106. 10.5852/ejt.2020.720.1117

[B6] Folmer O (1994) DNA primers for amplification of mitochondrial cytochrome c oxidase subunit I from metazoan invertebrates. Molecular Marine Biology and Biotechnology 3(5): 294–299. 7881515

[B7] Jones YL Peters SM Weland C Ivanova NV Yancy HF (2013) Potential use of DNA Barcodes in regulatory science: Identification of the U.S. food and drug administration’s “Dirty 22,” contributors to the spread of foodborne pathogens. Journal of Food Protection 76(1): 144–149. 10.4315/0362-028X.JFP-12-168 23317871

[B8] Kimura M (1980) A simple method for estimating evolutionary rates of base substitutions through comparative studies of nucleotide sequences. Journal of Molecular Evolution 16(2): 111–120. 10.1007/BF01731581 7463489

[B9] Kumar S Stecher G Tamura K (2016) MEGA7: Molecular Evolutionary Genetics Analysis Version 7.0 for Bigger Datasets. Molecular Biology and Evolution 33(7): 1870–1874. 10.1093/molbev/msw054 27004904 PMC8210823

[B10] Lanfear R Frandsen PB Wright AM Senfeld T Calcott B (2017) PartitionFinder 2: New methods for selecting partitioned models of evolution for molecular and morphological phylogenetic analyses. Molecular Biology and Evolution 34: 772–773. 10.1093/molbev/msw260 28013191

[B11] Li XR Zheng YH Wang CC Wang ZQ (2018) Old method not old-fashioned: Parallelism between wing venation and wing-pad tracheation of cockroaches and a revision of terminology. Zoomorphology 137(4): 519–533. 10.1007/s00435-018-0419-6

[B12] McKittrick FA (1964) Evolutionary studies of cockroaches. Cornell University Agricultural Experiment Station Memoir 389: 1–197.

[B13] Nguyen LT Schmidt HA von Haeseler A Minh BQ (2015) IQ-TREE: A fast and effective stochastic algorithm for estimating maximum-likelihood phylogenies. Molecular Biology and Evolution 32(1): 268–274. 10.1093/molbev/msu300 25371430 PMC4271533

[B14] Roth LM (1990) Revisionary studies on Blattellidae (Blattaria) from the Indo-Australian region. Memoirs of the Queensland Museum 28: 597–663.

[B15] Shiraki T (1931) Orthoptera of the Japanese Empire 2 (Blattidae). Insecta Matsumurana 5(4): 171–209. http://hdl.handle.net/2115/9222

[B16] Yang R Wang Z Zhou Y Wang Z Che Y (2019) Establishment of six new *Rhabdoblatta* species (Blattodea, Blaberidae, Epilamprinae) from China. ZooKeys 851: 27–69. 10.3897/zookeys.851.31403 31205442 PMC6557905

[B17] Yue QY Wu KL Qiu DY Hu J Liu DX Wei XY Chen J Cook CE (2014) A formal re-description of the cockroach *Hebardinaconcinna* anchored on DNA Barcodes confirms wing polymorphism and identifies morphological characters for field identification. PLoS ONE 9(9): e106789. 10.1371/journal.pone.0106789 PMC4169431 25232993

[B18] Zhu J Zhang JW Luo XX Wang ZQ Che YL (2022) Three cryptic *Anaplecta* (Blattodea, Blattoidea, Anaplectidae) species revealed by female genitalia, plus seven new species from China. ZooKeys 1080: 53–97. 10.3897/zookeys.1080.74286 35068964 PMC8752576

